# Comparing Elder and Child Abuse Prevention Approaches in Japan: Strategies for Reducing Elder Abuse

**DOI:** 10.1093/geroni/igaa057.150

**Published:** 2020-12-16

**Authors:** Asako Katsumata, Noriko Tsukada

**Affiliations:** 1 Rehabilitation Institution, Chiba, Tokyo, Japan; 2 Nihon University, Tokyo, Tokyo, Japan

## Abstract

This paper examines trends in elder abuse cases (types of abuse, traits of abusers, victims and their relationships, levels of disabilities and dementia of victims, etc.) by using longitudinal data (2012-2018) collected by Japan’s Ministry of Health, Labour and Welfare since its enforcement. Considering these trends, this paper then compares the Elder Abuse Prevention Law to the Child Abuse Prevention Law to assess differences in policy and program provisions and how those relate to successful prevention outcomes. For example, while reports for both elder abuse and child abuse cases have been increasing, governmental actions taken in response have varied. The Child Abuse Prevention Law has been modified 6 times since its enactment based on abuse cases, but no amendments have been made for Elder Abuse Prevention Law based on case or evaluation data. Moreover, there have been many public awareness campaigns for child abuse prevention, but none for elder abuse prevention. These efforts appear to have positive outcomes including increased reporting of child abuse to police. This analysis aims to compare abuse data, abuse laws and public health efforts for children and older adults in Japan. Findings seek to identify disparities and areas where the public approach to child abuse can inform and strengthen elder abuse policies and programs.

